# Stroma AReactive Invasion Front Areas (SARIFA) proves prognostic relevance in gastric carcinoma and is based on a tumor–adipocyte interaction indicating an altered immune response

**DOI:** 10.1007/s10120-023-01436-8

**Published:** 2023-10-24

**Authors:** Bianca Grosser, Christian M. Heyer, Johannes Austgen, Eva Sipos, Nic G. Reitsam, Andreas Hauser, Alison VanSchoiack, David Kroeppler, Dmytro Vlasenko, Andreas Probst, Alexander Novotny, Wilko Weichert, Gisela Keller, Matthias Schlesner, Bruno Märkl

**Affiliations:** 1 Pathology, Medical Faculty Augsburg, Institute of Pathology and Molecular Diagnostics, University of Augsburg, Stenglinstraße 2, 86156 Augsburg, Germany; 2 Biomedical Informatics, Data Mining and Data Analytics, Faculty of Applied Computer Science and Medical Faculty, University of Augsburg, Augsburg, Germany; 3 NanoString Technologies, Seattle, WA USA; 4 General and Visceral Surgery, Faculty of Medicine, University of Augsburg, Augsburg, Germany; 5 Gastroenterology, Faculty of Medicine, University of Augsburg, Augsburg, Germany; 6 Department of Surgery, TUM School of Medicine, Technical University of Munich, Munich, Germany; 7 Institute of Pathology, TUM School of Medicine, Technical University of Munich, Munich, Germany

**Keywords:** Gastric cancer, Stroma Areactive Invasion Front Areas (SARIFA), Biomarker, Histopathology, Molecular mechanisms

## Abstract

**Background:**

Recently, we presented Stroma AReactive Invasion Front Areas (SARIFA) as a new histomorphologic negative prognostic biomarker in gastric cancer. It is defined as direct contact between tumor cells and fat cells. The aim of this study was to further elucidate the underlying genomic, transcriptional, and immunological mechanisms of the SARIFA phenomenon.

**Methods:**

To address these questions, SARIFA was classified on H&E-stained tissue sections of three cohorts: an external cohort (*n* = 489, prognostic validation), the TCGA-STAD cohort (*n* = 194, genomic and transcriptomic analysis), and a local cohort (*n* = 60, digital spatial profiling (whole transcriptome) and double RNA in situ hybridization/immunostaining of cytokines).

**Results:**

SARIFA status proved to be an independent negative prognostic factor for overall survival in an external cohort of gastric carcinomas. In TCGA-STAD cohort, SARIFA is not driven by distinct genomic alterations, whereas the gene expression analyses showed an upregulation of FABP4 in SARIFA-positive tumors. In addition, the transcriptional regulations of white adipocyte differentiation, triglyceride metabolism, and catabolism were upregulated in pathway analyses. In the DSP analysis of SARIFA-positive tumors, FABP4 and the transcriptional regulation of white adipocyte differentiation were upregulated in macrophages. Additionally, a significantly lower expression of the cytokines IL6 and TNFα was observed at the invasion front.

**Conclusions:**

SARIFA proves to be a strong negative prognostic biomarker in advanced gastric cancer, implicating an interaction of tumor cells with tumor-promoting adipocytes with crucial changes in tumor cell metabolism. SARIFA is not driven by tumor genetics but is very likely driven by an altered immune response as a causative mechanism.

**Supplementary Information:**

The online version contains supplementary material available at 10.1007/s10120-023-01436-8.

## Introduction

Gastric cancer is ranked as the fifth most common cancer worldwide, accounting for approximately 769,000 cancer-associated deaths in 2020 [1]. Compared with other cancer entities, reliable and accepted biomarkers are sparse and currently restricted to microsatellite instability (MSI)-, Her2-, and PD-L1-analysis. The use of two established molecular classification systems is restricted to scientific analyses and has not yet entered routine diagnostics [2, 3].

Recently, we presented Stroma AReactive Invasion Front Areas (SARIFA) as a new histological prognostic marker in gastric and colon carcinomas. We defined SARIFA on hematoxylin and eosin (H&E)-stained tissue sections as the direct contact between the cluster of tumor glands/cells comprising at least five tumor cells and the inconspicuous surrounding adipose tissue at the invasion front. In our initial local study, SARIFA was found to be a negative independent prognostic factor for overall survival, with low inter-observer variability and minimal cost and time effort [4, 5].

The striking feature of SARIFA is the direct contact between tumor cells and adipocytes. Interestingly, Wulczyn et al. [6] and Foersch et al. [7] already identified this phenomenon recently as relevant in image analyses using artificial intelligence. There is substantial evidence for the existence of a tumor-promoting effect caused by crosstalk between tumor cells and cancer-associated adipocytes (CAAs), which has been investigated and reviewed by several groups and authors [8, 9]. An important effect of the crosstalk with CAAs is a change in the metabolism of tumor cells, including cellular energy production, which results in several tumor-promoting effects [8, 10]. Moreover, CCAs serve as an exogenous source of fatty acids, which are necessary for membrane synthesis and mitochondrial energy production in tumor cells [11].

Based on our previous results, where we observed that tumor cells adjacent to fat cells upregulate the fatty acid metabolism combined with higher macrophage counts, and based on fundamental research from the field of obesity research, we hypothesized that the morphological SARIFA phenomenon is potentially caused by an altered immune response to the tumor, enabling the direct contact of the tumor with tumor-promoting adipocytes and an adverse clinical course.

**Fig. 1:**
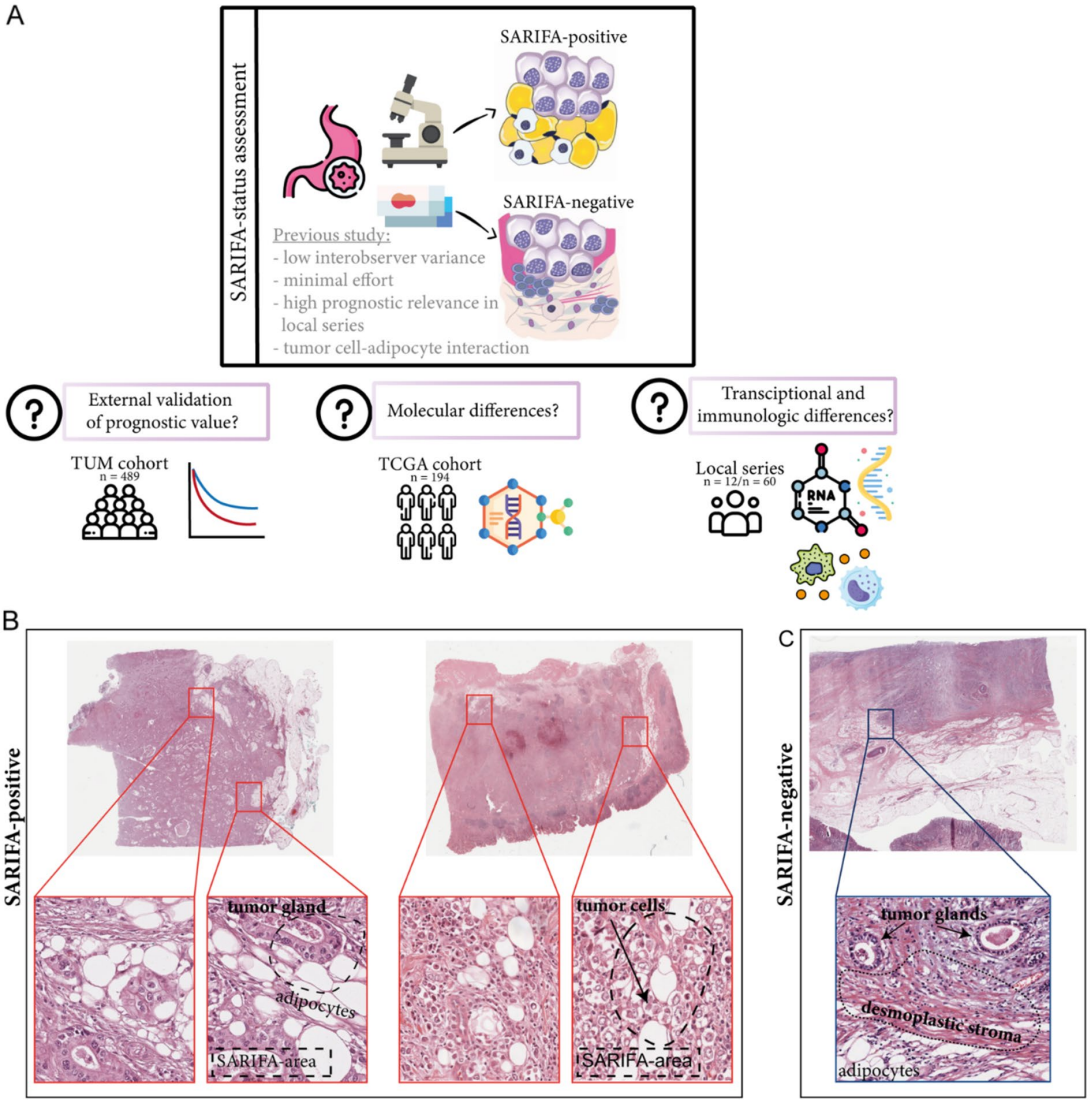
**A** Overview of the study design with results of previous studies and study aims. **B**, **C** Hematoxylin & Eosin-stained images of SARIFA-positive and SARIFA-negative cases of the TCGA-STAD cohort. **B** SARIFA-positive cancers of TCGA-STAD patients showing tumor cells directly adjacent to adipocytes without a stromal reaction. **C** SARIFA-negative cancer from a TCGA-STAD patient treated by surgery alone showing a desmoplastic reaction between tumor and fat at the invasion front

The aims of the study were to validate the prognostic relevance of SARIFA status in an independent external collection of adenocarcinomas of the stomach and gastroesophageal junction and to investigate the underlying genomic, transcriptional, and immunologic mechanisms of the SARIFA phenomenon (see Fig. 1A).

## Methods

### Definition and assessment of SARIFA

As described recently, SARIFA was evaluated using whole H&E-stained slide sections. We define SARIFA as an area located at the invasion front (IF) in which a tumor gland or a group of at least five tumor cells directly approach adipocytes without separating stroma. We classified tumors that presented these characteristics as SARIFA-positive (SARIFA) and the others as SARIFA-negative (non-SARIFA). Even if only a single SARIFA area was present, we classified the tumor as SARIFA-positive [4, 5]. In pT2 tumors, SARIFA-positive areas occur at the lateral invasion front in the submucosa or focal at perivascular adipocytes in the muscularis propria (see Supplementary Fig. S1). The first author (BG) assessed all tumors and did not have access to the clinical data. An exemplary preselected tumor slide was used for assessment in the external validation cohort. Example images for TCGA-STAD cohort are presented in Fig. 1B.

### Patients in the external validation cohort (TUM cohort)

The external validation cohort comprised surgical resection specimens from 489 patients with adenocarcinomas of the stomach and the gastroesophageal junction (AEG II and III, according to Siewert and Stein [12]) that at least presented infiltration of the submucosa (pT1b). These patients were treated between 2001 and 2012 at the Department of Surgery of the Technical University of Munich. The cohort has already been described in detail in recent studies [13]. All patients were treated with chemotherapeutic regimes based on platinum/5-fluorouracil (5FU). Furthermore, all surgical approaches included an abdominal D2 lymphadenectomy [13, 14]. Detailed clinicopathological characteristics are summarized in Supplementary Table S1.

The primary endpoint of the study was overall survival (OS), which was defined as the time elapsed between the date of diagnosis and the death of the patient due to any cause. The study was approved by the ethical committee of the Technical University of Munich (reference: 502/15s) and was performed in accordance with the Declaration of Helsinki.

### The Cancer Genome Atlas Stomach Adenocarcinoma (TCGA-STAD) cohort

The subset of The Cancer Genome Atlas Stomach Adenocarcinoma (TCGA-STAD) cohort, analyzed in the present study, comprised surgical resection specimens from 194 patients with adenocarcinomas of the stomach. Clinical data were downloaded from the cBioportal website (http://www.cbioportal.org/) [15]. H&E-stained whole slide images (named Dx) were downloaded from TCGA GDC Data Portal and assessed for SARIFA status [16]. Only the cases that presented adipose tissue on the slide were used for the assessment of SARIFA status (Fig. 1B). To assess the distribution of normal adipose tissue (adipose tissue in 16.5% of SARIFA-positive versus 15% of SARIFA-negative cases), the fresh frozen sections used for molecular analyses (named TS1 and BS1) were reviewed [2]. Detailed clinicopathological characteristics are shown in Supplementary Table S2.

### TCGA-STAD data analysis

Information regarding SARIFA status was added as a custom genetic track to the cBio Cancer Genomics Portal and genomic data analysis, and visualization was conducted in cBioportal. Details can be found in the Supplementary Methods.

### RNA in situ hybridization: RNA scope

Double in situ hybridization/immunostaining was performed on whole slide tissue sections of SARIFA-positive (*n* = 30) and SARIFA-negative (*n* = 30) cases, whose detailed patient characteristics have been described recently [5], using ACD Bio RNAscope probes (Advanced Cell Diagnostics, Newark, CA, USA) for Il-6 (Cat N 310378), Il-10 (Cat N 602058), Il-12 (Cat N 402068), and TNF alpha (Cat N 310428). Simultaneous immuno-histochemical staining with CD68 (KP-1, Cell Marque (Rocklin, USA), 1:200) allowed identification of the macrophages. Automated staining was performed using the RNAscope 2.5 assay (Cat N 322100) according to the manufacturer’s protocol on a Leica Bond RX staining system (Leica Microsystems, Wetzlar, Germany). PPIB (Cat. N 312028) and DapB (Cat N 312038) were used as positive and negative control probes for each run. Only strong distinct staining was assessed as a positive signal and quantified in four categories: very low, low, high, and very high. Staining was evaluated by the author JA for tumor center (TC), invasion front (IF), adipose tissue, and endothelium. Exemplary and additional difficult cases were evaluated by a pathologist (BG) and a board-certified pathologist (BM).

### NanoString’s GeoMx digital spatial profiling (DSP)

Using digital spatial profiling (DSP), we performed a multiplexed and spatially resolved profiling analysis for exemplary SARIFA-positive (*n* = 6) and SARIFA-negative (*n* = 6) cases on tissue microarrays (TMAs) (52 ROIS/2 slides), whose detailed patient characteristics have been described recently [5]. Details can be found in the Supplementary Methods. [17]

### Statistical analysis

Chi-squared or Fisher’s exact tests were used for the hypothesis testing of differences between relative frequencies. Continuous variables were compared using the Wilcoxon rank-sum test. Kaplan–Meier estimates of survival rates were compared using log-rank tests, and relative risks were estimated using hazard ratios (HR) obtained from Cox proportional hazard models. The median follow-up was calculated using the inverse Kaplan–Meier method [18]. Multiple testing corrections were performed using the Benjamini–Hochberg method. Statistical analyses were performed using SPSS Version 24 (IBM Corp., Armonk, NY, USA) and R Version 4.1.2. Exploratory significance levels (two-tailed) of 5% were used for hypothesis testing.

## Results

### SARIFA status and survival in an external validation cohort (TUM cohort)

In the external validation collection, the mean age of the patients was 64.6 years (range: 28.3–90.0 years), and the median follow-up time was 55.1 months (47.9–62.3 months). In total, 48% of the patients died during the follow-up period. The detailed clinico-pathological characteristics are summarized in Supplementary Table 1. Overall, 187 (38%) cases were classified as SARIFA-positive, and 302 (62%) were classified as SARIFA-negative.

**Fig. 2:**
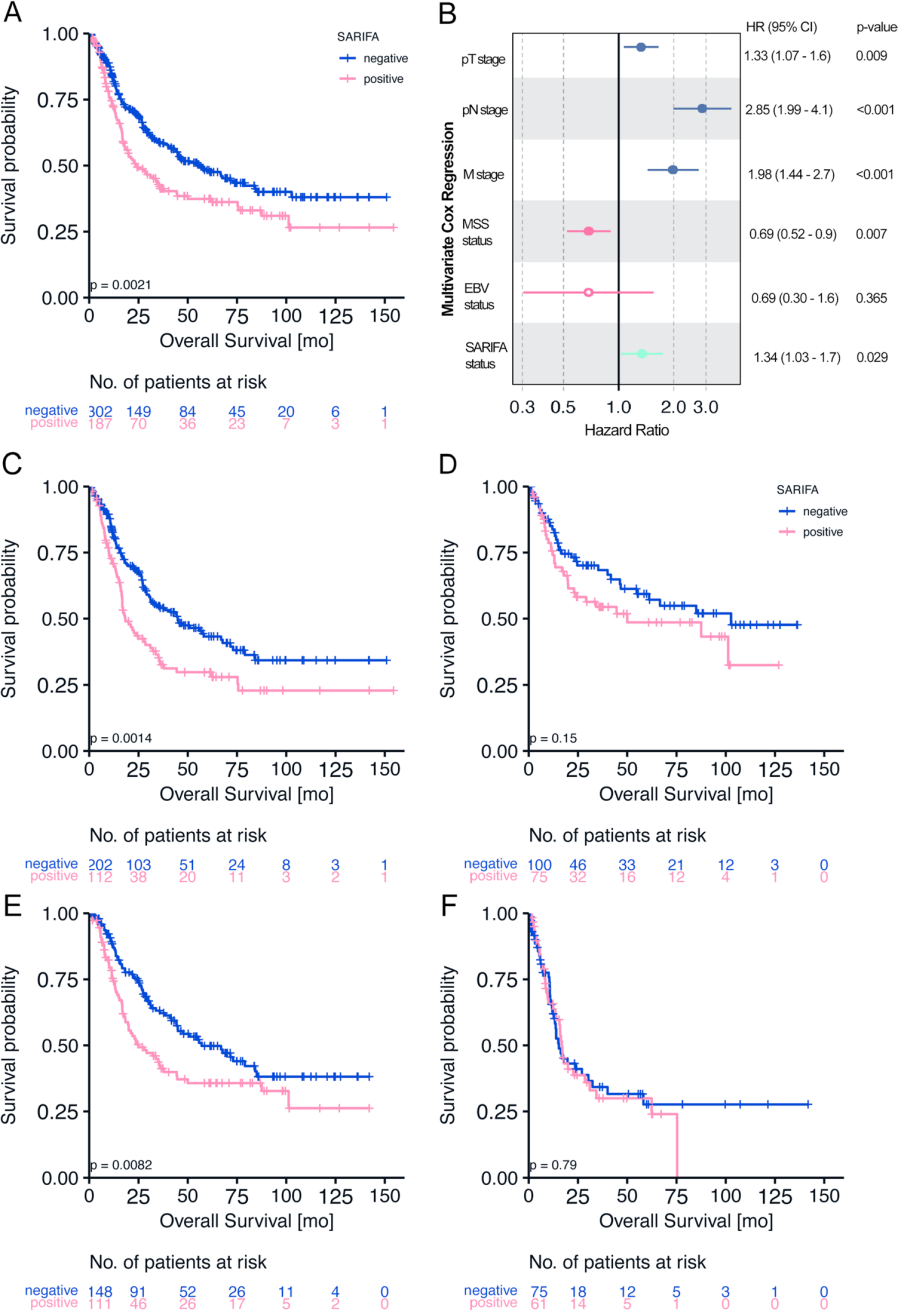
SARIFA and Survival: **A** Kaplan–Meier analysis of all TUM patients shows that patients with SARIFA-negative tumors have a significantly better survival (HR 1.498, 95% CI (1.156–1.940), *p* = 0.002). **B** Forest Plot of multivariate Cox Regression analysis including SARIFA status and known prognostic factors **C** Kaplan–Meier analysis of TUM patients treated with neoadjuvant chemotherapy shows that patient with SARIFA-negative tumor have a significantly better survival (HR 1.644, 95% CI (1.207–2.239), *p* = 0.002) **D** Kaplan–Meier analysis of TUM patients treated by surgery alone suggests improved survival in patients with SARIFA-negative tumors. However, the difference is statistically not significant (HR 1.425, 95% CI (0.882–2.302, *p* = 0.148)** E** Kaplan–Meier analysis of pT3 TUM patients shows that patient with SARIFA-negative tumor have a significantly better survival (HR 1.582, 95% CI (1.122–2.230), *p* = 0.009) **F** Kaplan–Meier analysis of pT4 TUM patients shows no significant survival difference of patients regarding SARIFA (HR 1.064, 95% CI (0.679–1.166), *p* = 0.788)

Patients with SARIFA-positive tumors had a significantly lower OS compared to SARIFA-negative patients (HR 1.498, 95% confidence interval (CI) (1.156–1.940), *p* = 0.002, Fig. 2A). The estimated median (95% CI) OS of patients with SARIFA-positive tumors was 23.8 months (13.9–33.7 months) compared to 55.7 months (38.6–72.8 months) for patients with SARIFA-negative tumors. Further, 56% of patients with SARIFA-positive tumors were dead at the end of the study period compared to 42% of patients with SARIFA-negative tumors.

Additionally, SARIFA positivity emerged as a statistically independent negative prognostic factor (HR 1.340, 95% CI (1.030–1.700), *p* = 0.029, Fig. 2B) for OS in Cox regression analyses, which were adjusted as per the known prognostic parameters (pT, pN, M, MSS, and EBV status).

The subgroup analysis indicated the existence of a negative prognostic effect of SARIFA in patients treated with neoadjuvant chemotherapy (HR 1.644, 95% CI (1.207–2.239), *p* = 0.002), which failed significance in surgery-only patients (HR 1.425, 95% CI (0.882–2.302, *p* = 0.148) (Fig. 2C, D). In the subgroup analysis regarding T stages, the prognostic effect of SARIFA was particularly seen in pT3 tumors (HR 1.582, 95% CI (1.122–2.230), *p* = 0.009) (Fig. 2E), while no significant survival differences were seen in pT4 tumors (HR 1.064, 95% CI (0.679–1.166), *p* = 0.788) (Fig. 2 F) and the other pT-stages (Supplementary Table S3). Further results of the subgroup analyses are summarized in Supplementary Table S3. In brief, the prognostic relevance of SARIFA status is observed in the subgroup with intestinal Laurén subtype and proximal tumor localization.

### Relationship between SARIFA status and clinicopathological characteristics

Clinicopathological characteristics for the TUM and TCGA-STAD cohorts stratified by SARIFA status are provided in Supplementary Tables S1 and S2.

In TUM patients, SARIFA positivity was associated with a higher depth of invasion (pT) (< 0.001), and patients were more likely to have regional lymph node metastasis (*p* = 0.008) and a positive R status (*p* = 0.001). In TCGA-STAD cohort, SARIFA-positive cases were associated with the presence of lymph node metastasis (*p* = 0.004).

### SARIFA status and survival in TCGA-STAD cohort

In TCGA-STAD cohort, the mean age of the patients was 65.5 years (range: 30.0–90.0 years), and the median follow-up time was 26.7 months (21.6–31.9 months). In total, 81 (42%) of the patients died during the follow-up period. Overall, 88 (46%) cases were classified as SARIFA-positive and 106 (54%) as SARIFA-negative. The detailed clinicopathological characteristics are summarized in Supplementary Table S2. Overall survival data were available for 190 (98%) patients.

In TCGA-STAD cohort, patients with SARIFA-positive tumors did not have significantly lower OS compared to SARIFA-negative patients (HR 1.206, 95% CI 0.776–1.876, *p* = 0.405, Supplementary Fig. S2A). The estimated median (95% CI) OS of patients with SARIFA-positive tumors was 25.6 months (17.6–33.7 months) compared to 42.5 months (18.1–67.0 months) for patients with SARIFA-negative tumors.

In addition, 40 (45%) patients with SARIFA-positive tumors died during the study period compared to 41 (39%) SARIFA-negative patients. Patients with SARIFA-negative tumors showed a trend toward better progression-free survival (HR 1.357, 95% CI 0.869–2.118, *p* = 0.179) and disease-free survival (HR 1.648, 95% CI 0.802–3.383, *p*  = 0.174) (Supplementary Fig. S2B, C).

### Genomic analyses in TCGA-STAD cohort

**Fig. 3:**
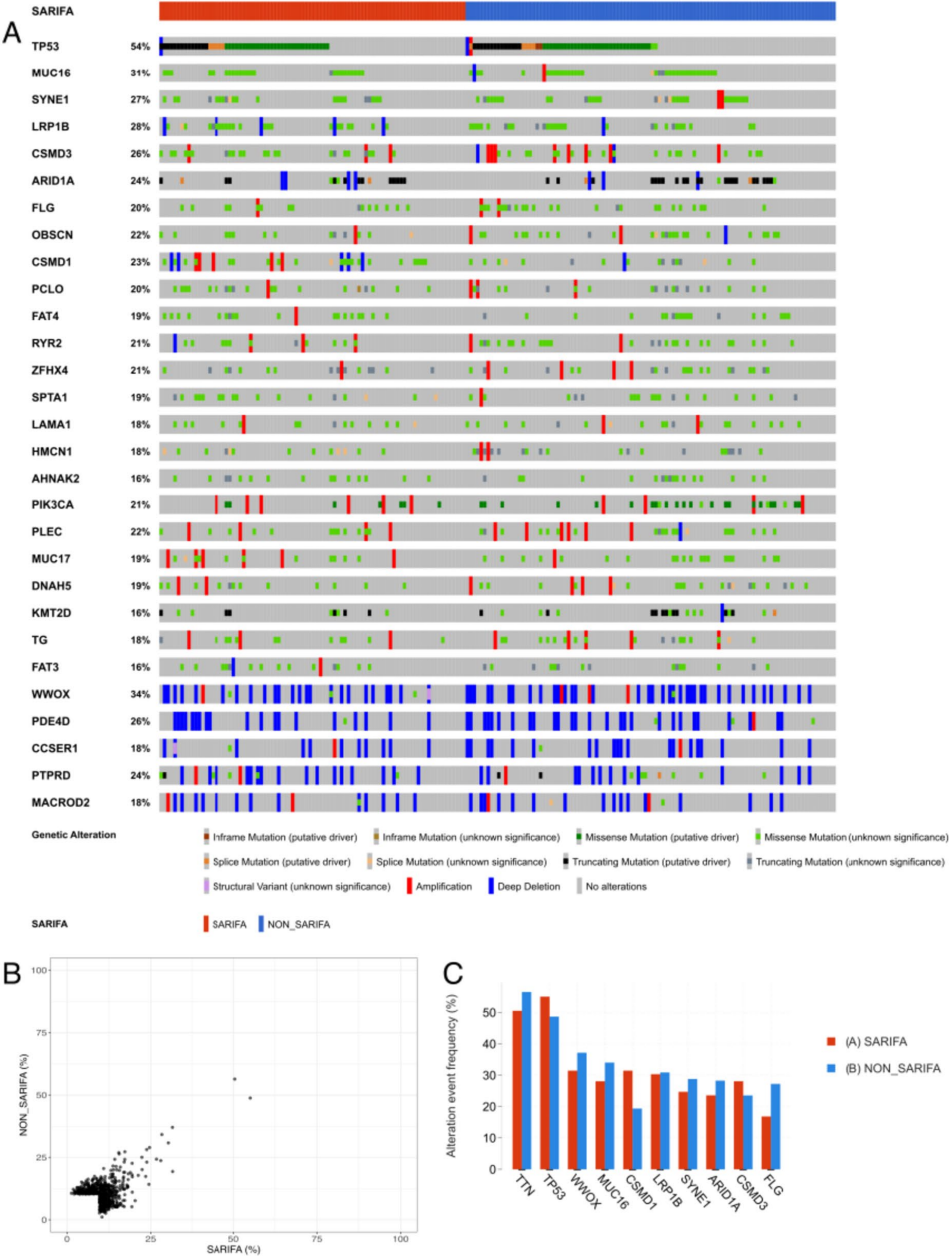
**A** Genomic analysis in the TCGA-STAD cohort: Oncoprint of top mutated genes for SARIFA-graded patients. Genes with mutations or CNVs in 15% of patients were included in the oncoprint. Genetic alterations were annotated from the OncoKB database. Samples are first split by SARIFA-class and then ordered based on the mutation occurrence. Genes are ordered by the occurrence frequency in the cohort. **B** Recurring genomic alterations are not exclusive to SARIFA groups. Comparison of percentage of samples with genomic alterations (SNVs, CNVs, SVs) in each SARIFA group. Only genomic alterations found in at least 10% of samples are shown. **C** Frequency of the most common genetic alterations in SARIFA and non-SARIFA samples

Next, we used data from TCGA-STAD cohort to investigate whether tumors bearing SARIFA characteristics have distinct genomic or transcriptomic properties. To check whether certain genomic alterations were more common in SARIFA-positive or SARIFA-negative tumors, the SARIFA classification rendered from the tissue slides was passed to the cBioPortal. An oncoprint of the genomics data shown in Fig. 3A shows the most common mutations found in TCGA-STAD cohort. Although a multitude of mutations and copy number variations were found, none of the alterations displayed immediately discernable patterns unique to one of the SARIFA groups. In Fig. 3B, the percentage of each genetic alteration in SARIFA and non-SARIFA samples is shown, with no alteration clearly associated with SARIFA status. As illustrated in Fig. 3C, the most recurrent alterations in included commonly mutated passenger genes, such as TTN and MUC16, independent of SARIFA status.

### Gene expression analysis in TCGA-STAD cohort

**Fig. 4:**
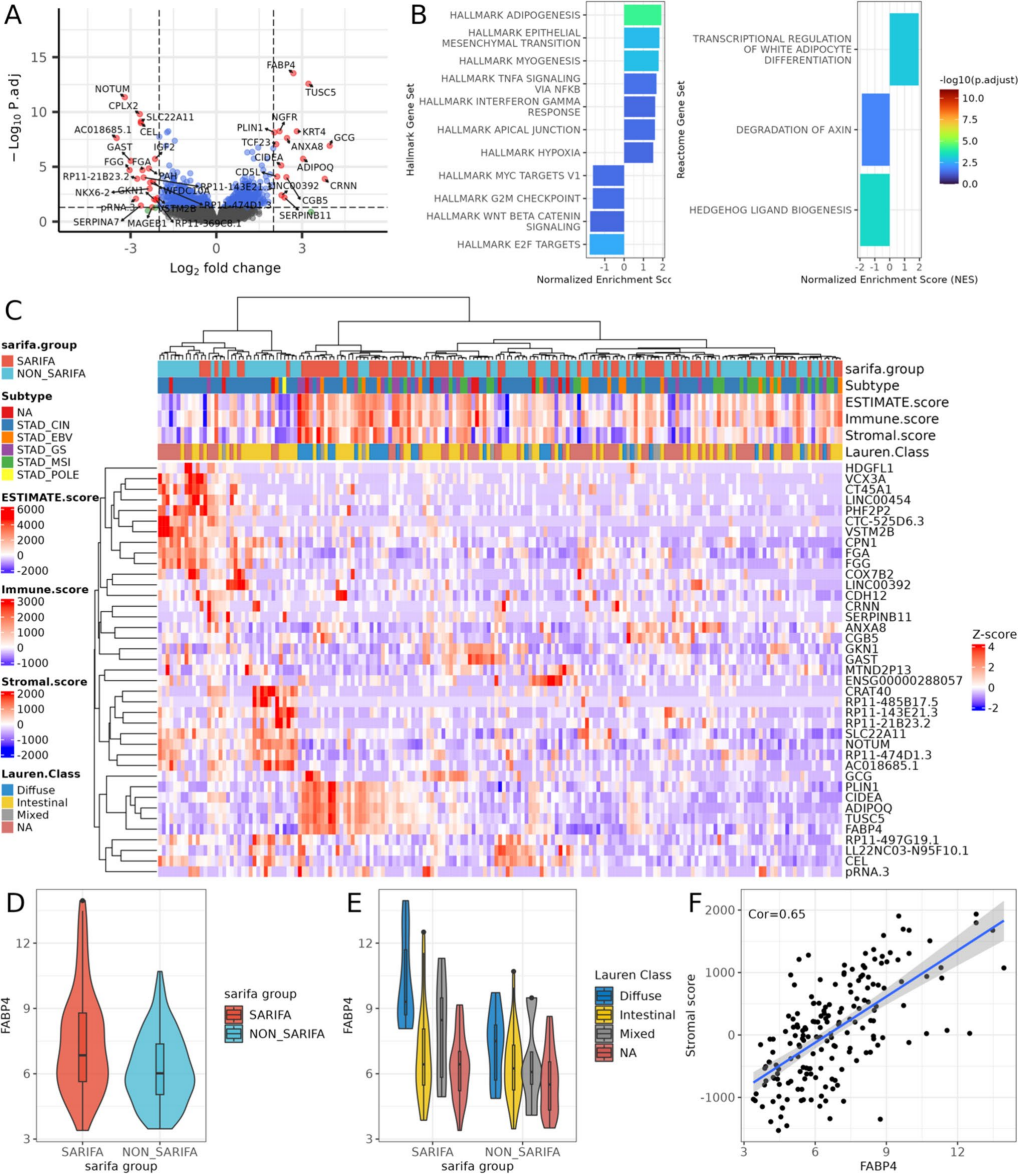
Bulk-transcriptome-analysis TCGA-STAD cohort. **A** Results of the differential expression analysis with DESeq2 between SARIFA and non-SARIFA samples. X-axis shows the Log2 fold change between the groups, y-axis, the –log10 of the adjusted *p* value from DESeq2. The dotted lines show Log2 fold change and adjusted *p* value thresholds at abs(2) and 0.05, respectively. **B** Results from GSEA from SARIFA vs non-SARIFA differential expression analysis. (left) Enrichments in MsigDB C2 Reactome gene sets; (right) Enrichments in MsigDB Hallmark gene sets. **C** Gene Expression heatmap of differentially expressed genes between SARIFA and non-SARIFA samples. Gene expression values were normalized with DESeq2 and then *z*-score transformed. The sample annotation rows illustrate various metadata parameters from TCGA-STAD cohort. Rows and Columns were clustered using Euclidean distance metric and Ward’s clustering. **D** Violin plot of normalized FABP4 expression in the TCGA-STAD cohort stratified by SARIFA status. Expression values are normalized with DEseq2's vst. **E** FAPB4 expression contrasting samples by Lauren's criteria classification, further stratified by SARIFA classification status. Expression values are normalized with DEseq2's vst. **F** Correlation between FABP4 normalized gene expression and Stromal score calculated for each sample. Correlation metric is Pearson’s correlation

Bulk transcriptomic data from TCGA were downloaded and analyzed using DESeq2. After matching SARIFA status with available gene expression data, 162 samples remained, consisting of 74 SARIFA and 88 non-SARIFA samples. Figure 4A shows the results from the DESeq2 analysis comparing SARIFA and non-SARIFA samples; 22 genes were found to be differentially expressed. The top genes overexpressed in the SARIFA samples (FABP4, TUSC5, ADIPOQ, and PLIN5) are associated with lipid transport and metabolism. Figure 4B illustrates the results of the GSEA analysis against the HALLMARK and REACTOME gene sets, where ADIPOGENESIS was enriched in the HALLMARK gene sets. In the REACTOME gene set enrichment, transcriptional regulation of white adipocyte differentiation was enriched in the SARIFA samples, whereas multiple MAP kinase-related pathways displayed downregulation in the SARIFA samples when compared to the non-SARIFA group.

To investigate whether the changes in gene expression between groups were driven by other processes, clinical metadata were annotated to gene expression patterns. Figure 4C illustrates that hierarchical clustering based on the expression levels of differentially expressed genes did not clearly group samples with the same SARIFA classification, similar ESTIMATE scores, or the same Lauren class.

Since transcriptomic changes between SARIFA groups seemed to be influenced by other clinical characteristics, the expression characteristics of FABP4 were investigated with respect to these factors. Figure 4D illustrates how the mean FAPB4 expression increased in the SARIFA-positive samples. When stratifying the samples based on Lauren’s criteria, SARIFA positivity was associated with increased FABP4 expression, especially in diffuse tumors. In the whole cohort, FAPB4 expression was correlated with the stromal score of the ESTIAMTE algorithm (Fig. 4F, Pearson’s correlation of 0.64).

### Spatially resolved transcriptome analysis in the tumor stroma and CD68 + cells

**Fig. 5:**
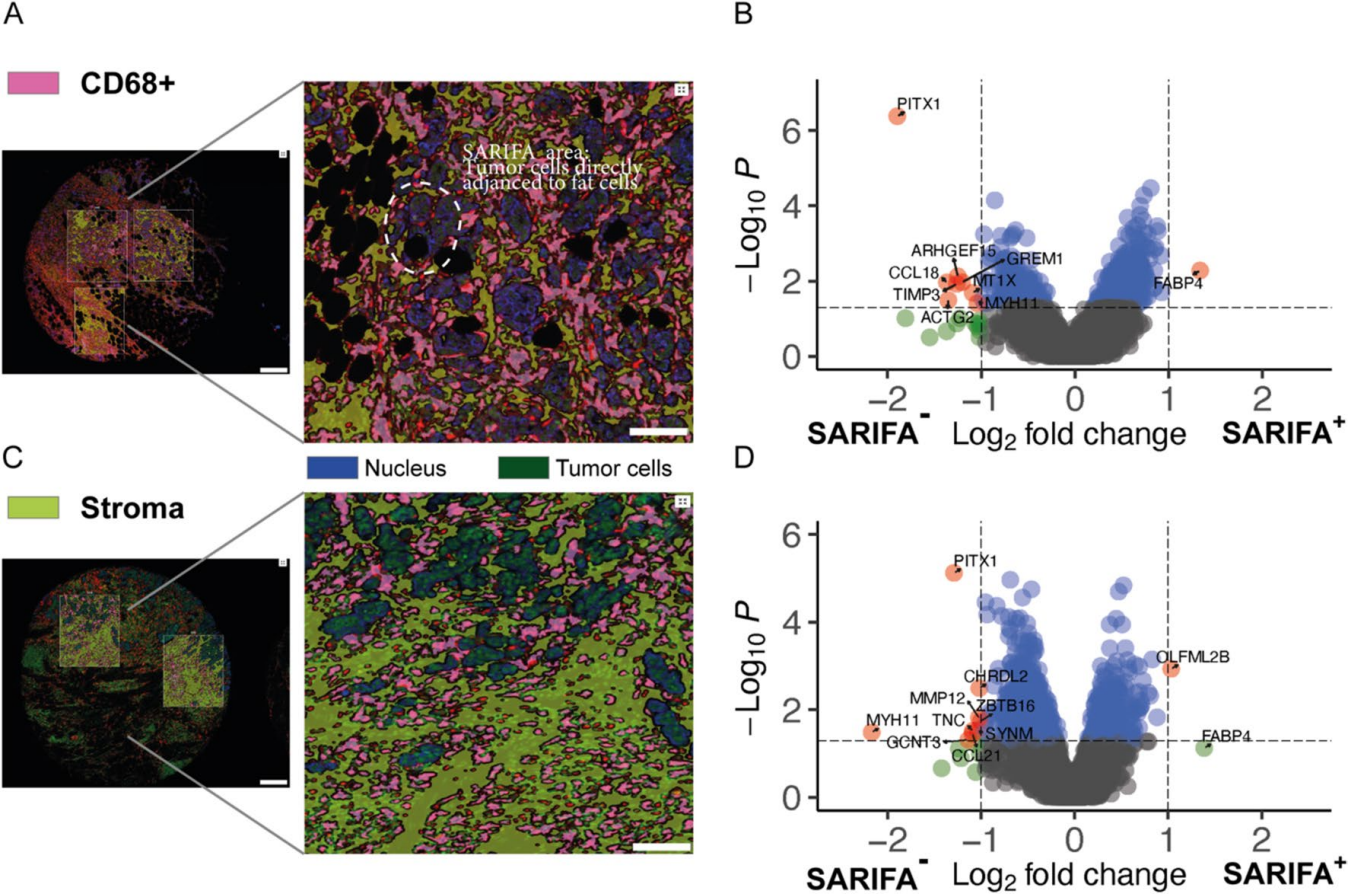
Digital spatial profiling analysis in CD68 + cells and Stroma: **A** Overview (scale bar 300 µm; ROIs chosen for multiplex profiling) and magnification (scale bar 100 µm; SARIFA area annotated) of a fluorescence image of a TMA core of an exemplary SARIFA-positive tumor visualizing the tumor cells, CD68 + cells and stroma. Within each region of interest, segmentation into different compartments is performed using CD68 fluorescent staining patterns as a mask to profile expression in CD68 + cells. **B** Volcano plot of the differential expression analysis in CD68 + cells between SARIFA-positive and negative samples. X-axis shows the Log2 fold change between the groups, y-axis the–log10 of the *p* value. The dotted lines show Log2 fold change and *p* value thresholds at abs(1) and 0.01, respectively. FABP4 is differentially upregulated in CD68 + cell in SARIFA-positive cases. **C** Overview (scale bar 300µm; ROIs chosen for multiplex profiling) and magnification (scale bar 100 µm) of a fluorescence image of a TMA core of an exemplary SARIFA-negative tumor visualizing the tumor cells, CD68 + cells and stroma. Within each region of interest, segmentation into different compartments is performed using ckpan fluorescent staining patterns as a mask to profile expression in the stroma component (ckpan negative). **D** Volcano plot of the differential expression analysis in the stroma between SARIFA-positive and negative samples. X-axis shows the Log2 fold change between the groups, y-axis the –log1010 of the *p* value. The dotted lines show Log2 fold change and *p* value thresholds at abs(1) and 0*·*01, respectively

Using DSP technology, we performed a whole transcriptome analysis in the stroma and in the subset of CD68 + cells, where we identified genes that were significantly upregulated at the invasion front (IF) in SARIFA-positive cases as compared to SARIFA-negative ones (Fig. 5).

In the stroma, the upregulated gene in SARIFA-positive cases at the IF was OLFML2B (Olfactomedin Like 2B). FABP4 showed a trend of higher expression in SARIFA-positive cases. In SARIFA-negative cases, PITX1, CHRDL2, ZBTB16, SYNM, MYH11, MMP12, TNC, CCL21, and GCNT3 were upregulated at the IF.

In the CD68 + cells in the SARIFA-positive cases at the IF, FABP4 was significantly upregulated, whereas in SARIFA-negative cases, PITX1, CCL18, TIMP3, GREM1, ACTG2, MYH11, MT1X, and ARHGEF15 were significantly higher expressed.

In the GSEA analysis against REACTOME gene sets, “the transcriptional regulation of white adipocyte differentiation,” “NGF-stimulated transcription,” and “chaperonin-mediated protein folding” were enriched in the macrophages in the SARIFA samples.

### SARIFA and expression of cytokines IL6, TNFα, IL10, and IL12

**Fig. 6:**
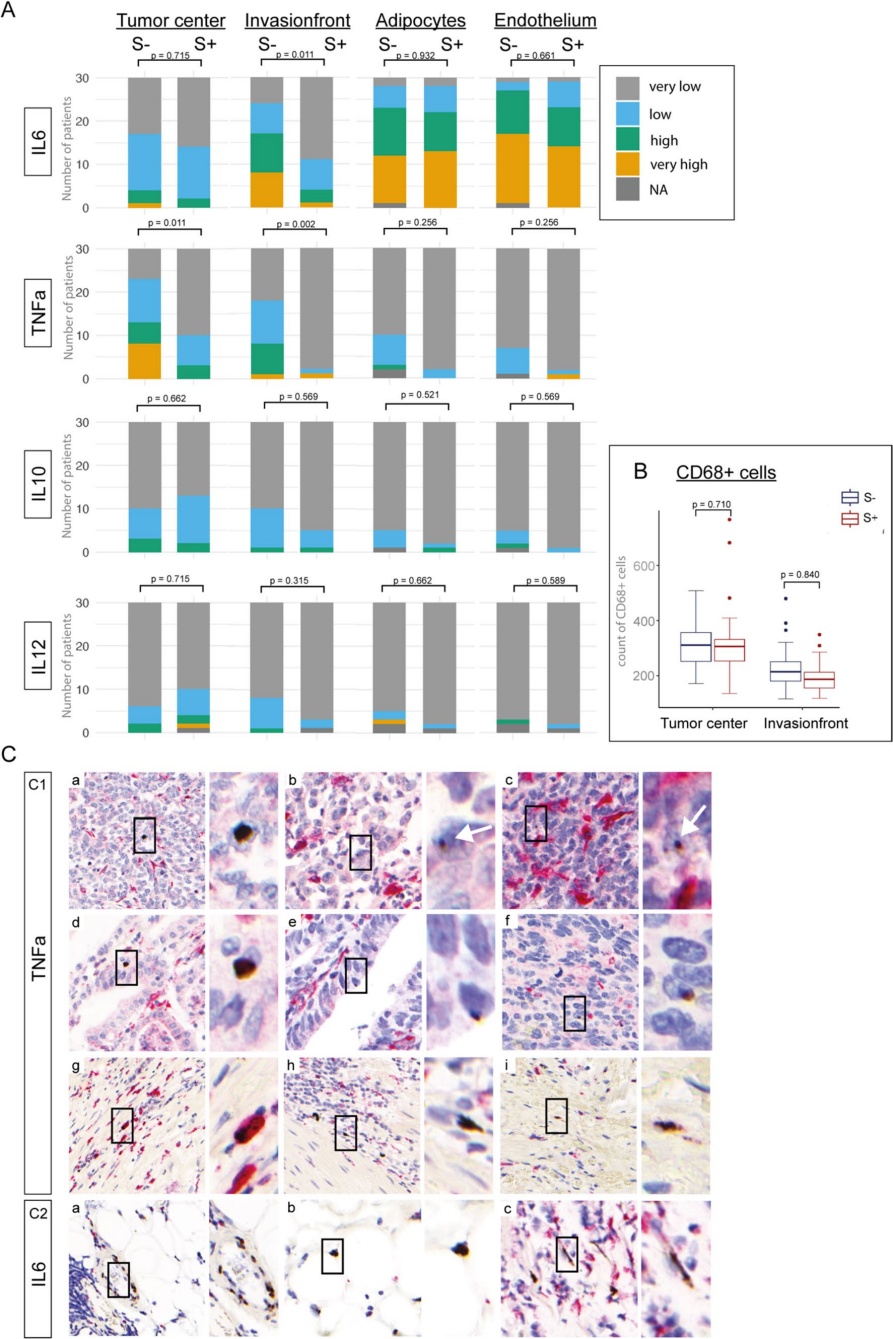
RNA Scope analysis: **A** Comparison of IL6, TNFα, IL10 and IL12 expression in regard to SARIFA status in the tumor center, invasion front, adipocytes and endothelium. *P*-values adjusted for multiple testing. **B** Comparison of macrophage count shows no difference in SARIFA-negative and -positive tumors in the tumor center and at the invasion front. **C** Double in situ hybridization/immunostaining images showing expression of TNFα and IL6 in brown as DAB-signal and CD68 + cells in red as fast-red signal (overview 400x; inserts 700x). **C1** a-f TNFα signal in tumor cells, C1 g CD68 + cells showing red staining and brown TNFα signal C1 h-i TNFα signal in spindle-like cells. **C2** a IL6 expression in endothelia of small capillaries, C2 b adipocytes, and C2 c spindle-like cells (most likely fibroblasts)

To address our hypothesis of an altered immune response, based on differential macrophage number in a previous study [5] and differential expression of cytokines by DSP, as the underlying mechanism of the SARIFA phenomenon, we performed a semi-quantitative analysis of the expression of cytokines using RNAScope. Interleukin 6 (IL6), tumor necrosis factor alpha (TNFα), and interleukin 12 (IL12) are potent immuno-stimulatory cytokines, whereas Interleukin 10 (IL10) represents a highly immunosuppressive cytokine [19]. Apart from being a major mediator of cancer-related inflammation, TNFα is involved in processes including cell survival and apoptosis [19–21]. In SARIFA-positive tumors, we observed a significantly lower expression of IL6 (*p* = 0.011) and TNFα (*p* = 0.002) at the IF. In addition, for SARIFA-positive cases, a significantly lower expression of TNFα was also observed in the tumor center (*p* = 0.011) compared to SARIFA-negative cases. In adipocytes and endothelia, there were no differences in IL6 and TNFα expression with respect to SARIFA status. IL10 and IL12 showed no significant differences in all localizations and cell types between SARIFA-positive and SARIFA-negative cases (Fig. 6A). There was no significant difference in the number of macrophages in the tumor center or at the IF (Fig. 6B).

The majority of TNFα signals in SARIFA-positive and SARIFA-negative tumors were found in the tumor cells (Fig. 6C1a-f). Few signals were also found in macrophages (Fig. 6C1g) and spindle-like cells, which most closely corresponded to fibroblasts (Fig. 6C1h, i). IL6 was expressed mainly in the endothelia of small capillaries (Fig. 6C2a), adipocytes (Fig. 6C2b), and spindle-like cells (most likely fibroblasts) (Fig. 6C2ca).

## Discussion

This study revealed five main results: (i) SARIFA proved to be a strong negative prognostic biomarker in in a large independent external cohort; (ii) as hypothesized, the SARIFA phenomenon is not driven by tumor genetics; (iii) bulk transcriptome analysis directs into the activation of lipid metabolism associated genes; (iv) stroma restricted spatial transcriptome analysis highlights the upregulation of FABP4 in macrophages; and (v) Il6 and TNFα, which are potent immune stimulators, are down-regulated in SARIFA-positive cases, indicating a supposed immune-suppressing mechanism as relevant for the development of SARIFA.

SARIFA status proved to be an independent negative prognostic marker for OS in an external cohort of gastric carcinomas and adenocarcinomas of the gastroesophageal junction, especially in advanced tumor stages. Significance was not reached regarding the prognostic value of SARIFA when analyzing TCGA-STAD dataset. However, the median survival rates were almost identical to those of the external cohort; therefore, the short follow-up time can be assumed to be the reason for the lack of significance.

The results of the external cohort validate the data that we published in a local series and prove that SARIFA is a robust prognostic biomarker in gastric cancer. The reason for its prognostic relevance, mainly in chemotherapy-treated patients, is probably due to the composition of the patient cohort with less advanced T stages in surgery-only patients (31% vs. 14% pT1/2), since prognostic relevance (as seen in the subgroup analyses) is observed in these patients. As the majority of SARIFA cases are in advanced pT stage, SARIFA by its nature is mainly a prognostic marker in already advanced cases. In the TUM cohort, especially in the subgroup of pT3 tumors, no significant difference in survival was observed for pT4 tumors, which may be due to the very short overall survival of this subgroup (median OS pT3: 44.3 vs. pT4: 16.4 months). Thus, the prognostic relevance in pT4 tumors has to be further investigated.

Since TNM staging and MSI status have been the only relevant prognostic markers in gastric cancer thus far, the determination of SARIFA status, which involves minimal time and cost effort because it is determined on routine H&E-stained tissue slides, and a remarkably high inter-observer agreement, will help in better stratifying and personalizing prognosis and, thus, therapy regimes [4, 5]. Given the adverse prognosis of SARIFA-positive cases, an efficient personalized therapy regimen is needed.

Analysis of the Omics data from TCGA-STAD cohort revealed that single genomic alterations do not influence the SARIFA status of the tumor. This supports our hypothesis that the SARIFA phenomenon is not based on specific genomic alterations and is caused by other potentially transcriptomic, immunologic, or even epigenetic alterations, which also has implications for considerations regarding a therapeutic strategy.

By analyzing TCGA-STAD cohort, we observed that transcriptomic differences can be detected between SARIFA-positive and SARIFA-negative samples. The in-depth characterization of differentially expressed genes revealed an uptick in gene expression linked to the fat metabolism and triglyceride catabolism. No transcriptomic markers sufficient to classify SARIFA-positive and SARIFA-negative samples were identified from bulk sequencing. In addition, FABP4 expression was influenced by tumor stromal score and Lauren subtype. Thus, these results must be interpreted with caution, as bulk sequencing data cannot exclude the possibility that these changes are due to differences in the number of non-tumor cells in the samples. However, molecular analyses of TCGA cohort used a smaller tissue sample compared with the diagnostic slides, which almost exclusively contained tumor tissue [2], and the adipose tissue in the tumor environment was almost equally distributed between SARIFA-positive and SARIFA-negative cases (16.5% in SARIFA-positive versus 15% in SARIFA-negative cases); however, the results of TCGA bulk sequencing data are consistent with our previous findings of an upregulation of genes associated with triglyceride metabolism in the tumor cells of SARIFA areas [5].

In addition to the above-mentioned spatial analysis of tumor cells [5], in the present study, we performed a spatial transcriptomic analysis of the tumor stroma. Since we previously observed an increased number of tumor-associated macrophages (TAMs) in SARIFA-positive cases [5], we analyzed them as a subgroup.

Interestingly, FABP4 was the only differentially expressed gene in the macrophages in SARIFA-positive tumors. FABP4 is a membrane-associated protein that serves as a lipid chaperone with a supposed function for intracellular lipid transport to specific compartments [11, 22]. Fatty acids are used as an energy source for the tricarboxylic acid cycle and for the synthesis of structural lipids or signaling molecules [23]. Migration, invasion, and tumor growth are enhanced by increased FABP4 expression through a FABP4-promoted detachment of tumor cells through the extracellular matrix (ECM), for which fatty acid oxidation (FAO) is essential [24]. Data from several cell culture and animal model experiments identified FABP4 as a potent tumor promoter in ovarian, prostate, cholangiocellular, and hepatocellular cancer [24–28]. In addition, FABP4 represents a fundamental protein for the dialog between cancer-associated adipocytes (CAAs) and tumor cells in ovarian cancer [23, 24, 28].

CAAs are part of the tumor microenvironment (TME). CAAs release free fatty acids (FFA) and extracellular vesicles to supply tumor cells with the substrate for FAO. TAMs and myeloid-derived stem cells (MDSCs) are also influenced by the increased amount of lipids in the TME, either through increased fatty acid uptake or prostaglandin and leukotriene synthesis resulting in a prostaglandin-mediated immunosuppression, which can be reversed by FAO inhibition [23, 29].

Indeed, we see reduced expression of the immuno-stimulatory cytokines IL6 and TNFα at the IF of SARIFA-positive tumors, which indicates an immunosuppressive environment in these cases [19]. The question to which extend the reduction of these cytokines could lead to a lack of stromal response seen in SARIFA-positive cases needs to be elucidated by via experimental investigations. There are different lipid-driven pathways that could also be of relevance in the context of SARIFA. These pathways lead to an immunosuppressive function of macrophages, which are often classified as anti-inflammatory M2 macrophages [30]. In addition, our recent results for colon carcinomas show a significant reduction in the number of natural killer (NK) cells in SARIFA areas and in the peripheral blood of SARIFA-positive colon carcinoma patients [31].

In addition, pro-inflammatory cytokines, such as CCL21 and CCL18, are increasingly expressed in SARIFA-negative cases. Both CCL21 and CCL18 stimulate the chemotaxis of activated T cells and lymphocytes. GREM1 cytokine acts as an inhibitor of monocyte chemotaxis [32].

In summary, the underlying mechanism of the SARIFA phenomenon is likely to be a lipid-mediated immunosuppressive TME.

Furthermore, the other differentially expressed genes in SARIFA-negative cases are involved in extracellular matrix breakdown and tissue remodeling, such as MMP12, TNC, TIMP3, ZBTB16, and CHRDL2 [32]. These results are basically consistent with the increased remodeling of the extracellular matrix that occurs during the development of a desmoplastic stromal reaction. However, additional analyses at the protein level are required for further clarification.

Because SARIFA positivity is associated with an adverse clinical course, these tumors call for the inclusion of a specific treatment as part of precision medicine. As mentioned above, genomic aberrations are not suitable for such treatments. However, the supposed basic mechanism of adipocyte-driven tumor progression offers the possibility of a pharmacological intervention targeting an altered tumor cell metabolism. Metformin, for example, besides its standard indication for diabetes, is known for its tumor-preventive and tumor-suppressive effects [33, 34]. SARIFA can serve as a biomarker for predicting the response to metformin therapy in cancer. Several specific FABP4 inhibitors have been synthesized, particularly for the treatment of type 2 diabetes and atherosclerosis. BMS309403 has been systematically evaluated in in vitro and in vivo diabetic models and has shown high efficacy in treating diabetes and atherosclerosis [35, 36]. Given that FABP4 has been identified as a strongly upregulated gene with heightened protein expression, along with growing evidence of a tumor-promoting effect of this protein, FABP4 inhibitors, such as BMS309403, are of great interest. Besides FABP4, CD36 or the genes involved in FAO, such as Nur77 [37], YAP, and CYP7a [38] could serve as a potential target in SARIFA-positive cases, as suggested by experimental data [39, 40].

Our study has some limitations. The prognostic relevance of SARIFA status, in addition to this retrospective analysis, needs to be investigated in independent prospective trials, especially in the subgroup of pT4 tumors. Our transcriptional results comprise only a limited sample size and need to be validated in larger cohorts. Furthermore, the relevance of the other differentially expressed genes involved in tissue remodeling needs to be elucidated in further analyses. To further characterize the immunological differences with regard to SARIFA, multiplex immunohistochemistry should be performed. Additionally, our study provides first insights into the mechanistic and immunologic background of the SARIFA phenomenon and provides initial hints for a further assessment of these complex processes.

## Conclusion

SARIFA proves to be a strong negative prognostic biomarker, especially in advanced, gastric cancer, implicating an interaction of tumor cells with tumor-promoting adipocytes causing a tumor-promoting switch toward fatty acid metabolism. Obviously, genetics do not play a mechanistic role; however, first insights indicate an altered immune response as a potential causative mechanism.

### Supplementary Information

Below is the link to the electronic supplementary material.

Supplementary file1 (DOCX 2712 KB)
