# Supplementary material for: Stroma AReactive Invasion Front Areas (SARIFA) proves prognostic relevance in gastric carcinoma and is based on a tumor–adipocyte interaction indicating an altered immune response
Source: Gastric Cancer. 2023 Oct 24;27(1):72–85. doi: 10.1007/s10120-023-01436-8 (PMC10761465; doi:10.1007/s10120-023-01436-8)
Supplement: Supplementary file 1 — Supplementary file1 (DOCX 2712 KB) [file 10120_2023_1436_MOESM1_ESM.docx]

**SUPPLEMENTARY MATERIAL**

**Supplementary Tables:**

**Supplementary Table S1.** Clinicopathological characteristics of the external validation cohort (TUM-cohort)

| Variable |  | n = 489 |  | SARIFA-positive (n=187) |  | SARIFA-negative (n=302) |  | *p* value |
| --- | --- | --- | --- | --- | --- | --- | --- | --- |
| Median Age (range) [years] | | 64.6 (28 - 90) |  | 64.6 (29 - 91) |  | 64.4 (28 - 85) |  | 0.898 |
| Median Follow-up (95% CI) [mo] | | 55.1 (47.9 - 62.3) |  | 58.4 (42.6 - 74.2) |  | 54.6 (46.1 - 63.1) |  | 0.538 |
| Sex |  |  |  |  |  |  |  | 0.197 |
|  | female | 112 | 23% | 37 | 20% | 75 | 25% |  |
|  | male | 377 | 77% | 150 | 80% | 227 | 75% |  |
| pT category |  |  |  |  |  |  |  | <0.001 |
|  | pT1/2 | 94 | 19% | 15 | 8% | 79 | 26% |  |
|  | pT3/4 | 395 | 81% | 172 | 92% | 223 | 74% |  |
| pN category |  |  |  |  |  |  |  | 0.008 |
|  | negative | 144 | 29% | 42 | 22% | 102 | 34% |  |
|  | positive | 345 | 71% | 145 | 78% | 200 | 66% |  |
| Distant Metastasis | |  |  |  |  |  |  | 0.338 |
|  | no | 410 | 84% | 153 | 82% | 257 | 85% |  |
|  | yes | 79 | 16% | 34 | 18% | 45 | 15% |  |
|  |  |  |  |  |  |  |  |  |
| Grade of differentiation |  |  |  |  |  |  |  | 0.052 |
|  | low grade | 86 | 18% | 25 | 13% | 61 | 20% |  |
|  | high grade | 313 | 64% | 127 | 68% | 186 | 62% |  |
|  | NA | 90 | 18% |  |  |  |  |  |
| Lauren |  |  |  |  |  |  |  | <0.001 |
|  | intestinal | 276 | 56% | 86 | 46% | 190 | 63% |  |
|  | non-intestinal | 213 | 44% | 101 | 54% | 112 | 37% |  |
| Localization |  |  |  |  |  |  |  | 0.091 |
|  | proximal | 246 | 50% | 85 | 45% | 161 | 53% |  |
|  | non-proximal | 243 | 50% | 102 | 55% | 141 | 47% |  |
| R status |  |  |  |  |  |  |  | 0.001 |
|  | R0 | 364 | 74% | 124 | 66% | 240 | 79% |  |
|  | R1 | 125 | 26% | 63 | 34% | 62 | 21% |  |
| EBV status |  |  |  |  |  |  |  | 0.546 |
|  | EBV  negative | 466 | 95% | 176 | 94% | 290 | 96% |  |
|  | EBV  positive | 19 | 4% | 9 | 5% | 10 | 3% |  |
|  | NA | 4 | 1% |  |  |  |  |  |
| MMRD status |  |  |  |  |  |  |  | 0.788 |
|  | MSS | 440 | 90% | 167 | 89% | 273 | 90% |  |
|  | MSI | 45 | 9% | 18 | 10% | 27 | 9% |  |
|  | NA | 4 | 1% |  |  |  |  |  |
| Death |  |  |  |  |  |  |  | 0.003 |
|  | no | 256 | 52% | 82 | 44% | 174 | 58% |  |
|  | yes | 233 | 48% | 105 | 56% | 128 | 42% |  |
| nCTx |  |  |  |  |  |  |  | 0.117 |
|  | no | 175 | 36% | 75 | 40% | 100 | 33% |  |
|  | yes | 314 | 64% | 112 | 60% | 202 | 67% |  |
| TRG |  |  |  |  |  |  |  | 0.048 |
|  | 2 | 144 | 29% | 43 | 23% | 101 | 33% |  |
|  | 3 | 170 | 35% | 69 | 37% | 101 | 33% |  |
| Legend: p-values are shown for difference between SARFA-positive and SARIFA-negative tumors | | | | |  |  |  |  |
| Abbreviations: nCTx: neoadjuvant CTx, MMRD: mismatch repair deficiency; MSS: Microsatellite stable; MSI: Microsatellite instable; TRG: Tumor regression grade; NA: not available | | | | | | | | |
| **Supplementary Table S2.** Clinicopathological characteristics of the TCGA-STAD cohort | | | | | | | | |

|  |  | All cases  (n = 194) |  | SARIFA-positive  (n=88) |  | SARIFA-negative  (n=106) |  | *p* value |
| --- | --- | --- | --- | --- | --- | --- | --- | --- |
| Median Age (range) [years] | | 65.5 (30 - 90) |  | 63.5 (43 - 90) |  | 67.0 (30 - 90) |  | 0.123 |
| Median Follow-up (95% CI) [mo] | | 26.7 (21.6 – 31.9) |  | 26.4 (17.9 – 34.9) |  | 26.9 (21.0 – 32.9) |  | 0.642 |
| Sex |  |  |  |  |  |  |  | 0.403 |
|  | female | 71 | 37% | 35 | 40% | 36 | 34% |  |
|  | male | 123 | 63% | 53 | 60% | 70 | 66% |  |
| pT category |  |  |  |  |  |  |  | 0.874 |
|  | pT1/2 | 32 | 16% | 15 | 17% | 17 | 16% |  |
|  | pT3/4 | 161 | 83% | 73 | 83% | 88 | 83% |  |
|  | NA | 1 | 1% |  |  |  |  |  |
| pN category |  |  |  |  |  |  |  | 0.004 |
|  | negative | 57 | 29% | 17 | 19% | 40 | 38% |  |
|  | positive | 136 | 70% | 71 | 81% | 65 | 61% |  |
|  | NA | 1 | 1% |  |  |  |  |  |
| Distant Metastasis | |  |  |  |  |  |  | 0.144* |
|  | no | 101 | 52% | 79 | 90% | 101 | 95% |  |
|  | yes | 8 | 4% | 6 | 7% | 2 | 2% |  |
|  | NA | 85 | 44% |  |  |  |  |  |
| Grade of differentiation |  |  |  |  |  |  |  | 0.090 |
|  | low grade | 73 | 38% | 27 | 31% | 46 | 43% |  |
|  | high grade | 117 | 60% | 58 | 66% | 59 | 56% |  |
|  | NA | 4 | 2% |  |  |  |  |  |
| TCGA-subgroup |  |  |  |  |  |  |  | 0.074 |
|  | EBV+ | 17 | 9% | 5 | 6% | 12 | 11% |  |
|  | MMRD | 26 | 13% | 8 | 9% | 18 | 17% |  |
|  | GS | 22 | 11% | 14 | 16% | 8 | 8% |  |
|  | CIN | 104 | 54% | 48 | 55% | 56 | 53% |  |
|  | POLE | 1 | 1% | 0 | 0% | 1 | 1% |  |
|  | NA | 24 | 12% |  |  |  |  |  |
| Death |  |  |  |  |  |  |  | 0.341 |
|  | no | 113 | 58% | 48 | 55% | 65 | 61% |  |
|  | yes | 81 | 42% | 40 | 45% | 41 | 39% |  |
| nCTx |  |  |  |  |  |  |  |  |
|  | no | 194 | 100% | 88 | 100% | 106 | 100% |  |
|  | yes | 0 | 0% | 0 | 0% | 0 | 0% |  |
| Legend: p-values from Pearson’s chi squared test (*Fisher's exact test) are shown for difference between SARIFA-positive and SARIFA-negative tumors | | | | | | | |  |
| Abbreviations: nCTx: neoadjuvant CTx, TCGA: The Cancer Genome Atlas, EBV+: EBV positive, MMRD: mismatch repair deficiency; GS: genomic stable; CIN: chromosomal instable | | | | | | | | |

**Supplementary Table S3.** Subgroup analyses external validation cohort: univariate Cox regression analyses

|  |  | HR | CI |  | p value | p value log rank |
| --- | --- | --- | --- | --- | --- | --- |
| nCTx | no (n=175) | 1.425 | 0.882 | 2.302 | 0.148 | 0.150 |
|  | yes (n=314) | 1.644 | 1.207 | 2.239 | 0.002 | 0.001 |
| (y)pT | (y)pT1 (n=32) | 0.037 | 0.000 | 4635.884 | 0.583 | 0.385 |
|  | (y)pT2 (n=62) | 0.925 | 0.310 | 2.755 | 0.888 | 0.888 |
|  | (y)pT3 (n=259) | 1.582 | 1.122 | 2.230 | 0.009 | 0.008 |
|  | (y)pT4 (n=136) | 1.064 | 0.679 | 1.166 | 0.788 | 0.788 |
| Lauren | intestinal (n=276) | 1.554 | 1.087 | 2.222 | 0.016 | 0.015 |
|  | nicht-intestinal (n=213) | 1.389 | 0.944 | 2.046 | 0.096 | 0.094 |
| Localization | proximal (n=246) | 1.821 | 1.287 | 2.578 | 0.001 | 0.001 |
|  | non-proximal (n=243) | 1.317 | 0.890 | 1.950 | 0.168 | 0.167 |

Legend: Hazard Ratio of overall survival of SARIFA positive cases - Reference: SARIFA-negative cases; Legend: CI: Confidence interval (95%); nCTx: neoadjuvant CTx,

**Supplementary Figure:**

**
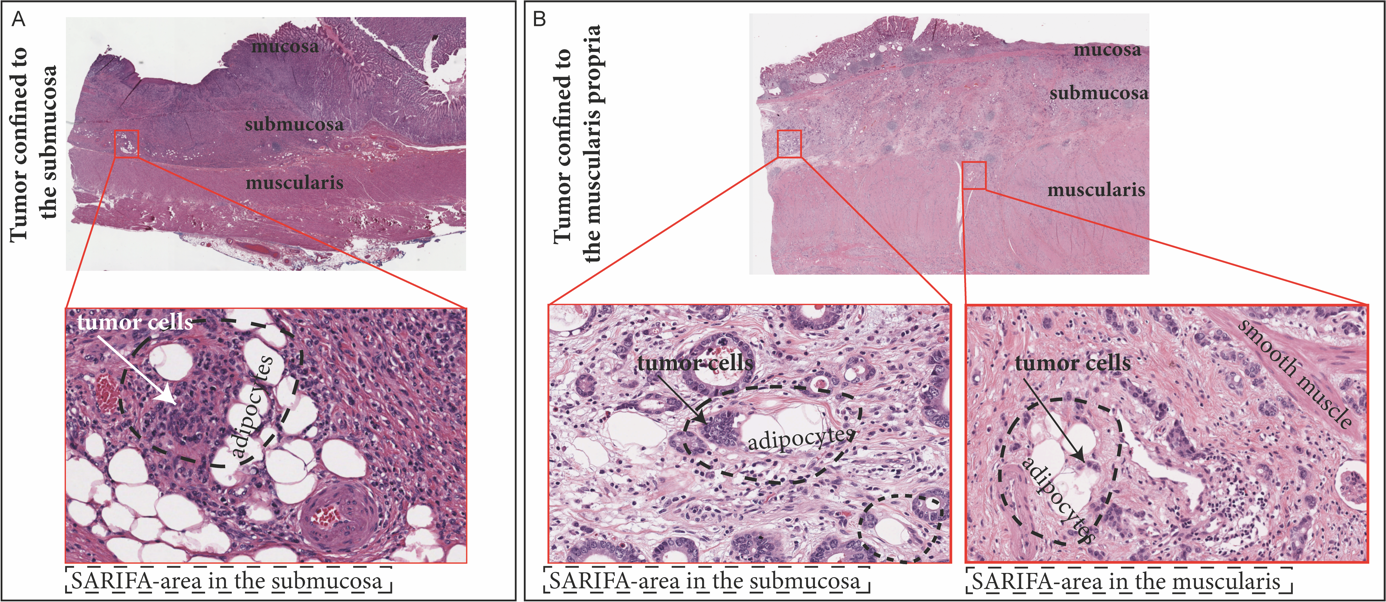
**

**Supplementary Figure S1: A:** SARIFA-positive cancer, confined to the submucosa, of a TCGA-STAD patients showing tumor cells directly adjacent to adipocytes without a stromal reaction (SARIFA-area) in the submucosa. **B:** SARIFA-positive cancer, confined to the muscularis propria, from a TCGA-STAD patient showing tumor cells directly adjacent to adipocytes (SARIFA-area) at the lateral invasion front in the submucosa and focal tumor cells adjacent to perivascular adipocytes in the muscularis propria.


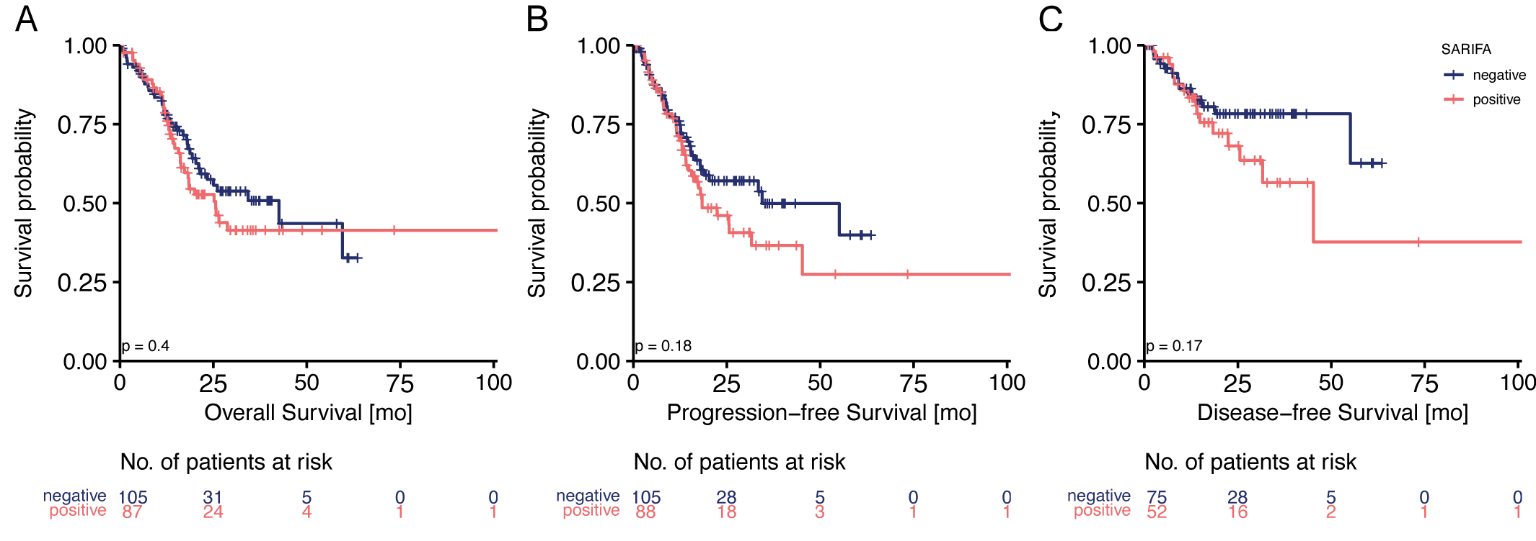


**Supplementary Figure S2:** Kaplan Meier analysis of TCGA-STAD patients: **A** patients with SARIFA-positive tumors did not have significantly lower OS compared to SARIFA-negative patients (HR 1.206, 95% CI 0.776-1.876, p = 0.405) **B, C** Patients with SARIFA-negative tumors showed a trend towards better progression-free survival (HR 1.357, 95% CI 0.869-2.118, p = 0.179) and disease-free survival (HR 1.648, 95% CI 0.802-3.383, p = 0.174).

**Supplementary Methods:**

***TCGA-STAD data analysis***

Information regarding SARIFA status was added as a custom genetic track to the cBio Cancer Genomics Portal and genomic data analysis, and visualization was conducted in cBioportal. This included generating oncoprints of recurrent genomic alterations and conducting Fisher’s exact tests on genomic alterations between SARIFA and non-SARIFA samples. The results of TCGA ESTIMATE analysis were downloaded from <https://bioinformatics.mdanderson.org/estimate>. The ESTIMATE algorithm generates the following three scores: stromal score (captures the presence of stroma in tumor tissue), immune score (represents the infiltration of immune cells in tumor tissue), and estimate score (infers tumor purity) ^41^. In addition, RNAseq count data and metadata from TCGA-STAD cohort were retrieved using the TCGAbiolinks package ^42,43^ and integrated with the metadata from cBioportal and the ESTIMATE results.

Gene expression count data were processed and normalized with DESeq2 using default parameters ^44^. For gene expression visualization, normalized expression counts were further normalized using variance-stabilizing transformation (VST) from DESeq2. Differential gene expression analysis was performed with DESeq2. Genes were considered differentially expressed at abs(Log2FoldChange)>2 and Benjamini–Hochberg-adjusted p-values <0.05. GSEA ^45^ against REACTOME and HALLMARK gene sets downloaded from MSigDB 7.5.1 were run and visualized using the clusterProfiler package ^46,47^. For gene set ranking, the following metric was used: −log10(p.value) * Log2FoldChange.

Gene expression heatmaps were generated using ComplexHeatmap ^48^. The VST-normalized counts from DESeq2 were further normalized with a z-score transformation per gene to visualize the expression differences between the samples.

All analyses were conducted using R version 4.2.1 and the Bioconductor 3.15 Docker container. The analysis scripts for reproducing the analysis are deposited at <https://github.com/schlesnerlab/sarifa-rnaseq>.

***NanoString’s GeoMx digital spatial profiling (DSP)***

Using digital spatial profiling (DSP), we performed a multiplexed and spatially resolved profiling analysis for exemplary SARIFA-positive (n=6) and SARIFA-negative (n=6) cases on tissue microarrays (TMAs) (52 ROIS/2 slides), whose detailed patient characteristics have been described recently ^5^. DSP technology uses RNA detection probes with ultraviolet (UV) photocleavable indexing oligos for transcriptomic profiling within the selected regions of interest (ROIs). Using the Leica Biosystems Bond RX FFPE RNA Slide Preparation protocol from NanoString (HIER 20 min with ER2 at 100°C, 1 ug/ml Proteinase K for 15 min), 5 µm-thick FFPE sections were prepared. Morphology markers for visualizing tumor cells (PanCK AF488, Novus NBP2-33200AF488, 1 μg/mL), CD68+ cells (CD68 AF647, Santa Cruz sc-20060AF647, 0.5 μg/mL), and Syto13 (ThermoFisher Scientific S7575) were applied for 1 hour at room temperature prior to being loaded on the GeoMx Digital Spatial Profiler. Based on fluorescence imaging and the corresponding H&E images, ROIs (200–600 µm in diameter) within the SARIFA-positive and SARIFA-negative areas were chosen for multiplex profiling. The DSP exposed each ROI to a 385-nm light to release the indexing oligos, and the photocleaved oligos were transferred into a microwell. In addition, sequencing libraries were generated as described previously ^17^, according to NanoString’s GeoMx-NGS Readout Library Prep instructions, and sequenced on Illumina NovaSeq. The DSP sequencing data were processed using the GeoMx NGS Pipeline (DND). Reads were trimmed, merged, and aligned to a list of indexing oligos to identify the source probe, and the unique molecular identifier (UMI) region of each read was used to remove PCR duplicates and duplicate reads, converting reads into digital counts. The 75th percentile of target counts within each ROI was calculated and normalized to the geometric mean of the 75th percentiles across all ROIs. The DSP data analysis was conducted using the GeoMx Analysis Suite Version 2.5.1.145 according to the GeoMx–DSP Data Analysis User Manual (MAN-10154, NanoString Technologies). In brief, the negative control probes were used to estimate the number of background counts at each data point. The Q3 normalization method was applied to the filtered data set (after the exclusion of genes expressed below the limit of quantification (LOQ); 13897 gene targets out of 18677 retained). The p values for differences in SARIFA status were calculated using a non-parametric t-test. GSEA analyses are performed against REACTOME gene sets using the GeoMx Analysis Suite under default settings.
